# In memoriam: Carl J. Carrano: July 14, 1950–January 26, 2022

**DOI:** 10.1007/s10534-024-00651-9

**Published:** 2025-03-12

**Authors:** Frithjof C. Küpper, Eleanor Carrano, Kenneth N. Raymond, Eric Miller, Ricardo Cruz-López, Ana Mijovilovich, Kyoko Yarimizu, Andrew Cooksy, Wes Harris, Shady A. Amin, Alison Butler, John C. Carrano

**Affiliations:** 1 School of Biological Sciences, University of Aberdeen, Cruickshank Building, St. Machar Drive AB24 3UU, Aberdeen, Scotland; 2 Marine Biodiscovery Centre, Department of Chemistry, University of Aberdeen, AB24 3UE, Aberdeen, Scotland; 3 Department of Chemistry and Biochemistry, San Diego State University, San Diego, CA 92182-1030 USA; 4 Formerly of Department of Earth and Environmental Sciences, San Diego State University, San Diego, CA 92182-1030, USA; 5 Department of Chemistry, University of California, Berkeley, CA 94720 USA; 6 Phytophile Consulting, Long Beach, CA 90803 USA; 7 Universidad Autónoma de Baja California (UABC), Instituto de Investigaciones Oceanológicas, Ensenada, Baja California 22830 México; 8 Czech Academy of Sciences, Biology Centre, Institute of Plant Molecular Biology, Laboratory of Plant Biophysics and Biochemistry, Branišovská 31/1160, 370 05, České Budějovice, Czech Republic; 9 Microbial Genomics and Ecology, The IDEC Institute, Hiroshima University, Higashihiroshima, Japan; 10 Department of Chemistry and Biochemistry, University of Missouri-St. Louis, St. Louis, MO 63,121 USA; 11 Marine Microbiomics Lab, Biology Program, New York University Abu Dhabi, Abu Dhabi, UAE; 12 Department of Chemistry and Biochemistry, University of California, Santa Barbara, CA 93106 USA; 13 Formerly with DARPA, Now Retired, 217 E 7 Oaks Dr, Greenville, SC 29605 USA; 14 Oceanography Center, University of Cyprus, 1 Panepistimiou Av., 2109 Aglandjia, Nicosia, Cyprus

**Keywords:** Iodine–iron, Rhodotorulic acid, Siderophores

## Abstract

This article is a celebration of the life and work of Carl J. Carrano who, from a childhood in Long Island, New York, built a career in bioinorganic chemistry, especially in the context of metal uptake and halogen metabolism in microbes and marine organisms.

## Frithjof C. Küpper

*Carl’s life, career and our friendship and collaboration about the inorganic biochemistry of marine algae.*

*(based upon 2 days of interviews with Eleanor Carrano in Khiran, Kuwait, April 2024).*

Carl Joseph Carrano was born in Rockville Centre, NY, on the 14th of July 1950 as the oldest child of Con and Eleanor Carrano. Carl’s father was Italian American, born in the United States to Italian immigrants. Carl’s mother, Eleanor, was mostly of German descent (Eleanor is also the name of Carl’s youngest daughter), including from the area that is close to Brno, now the Czech Republic. Carl’s mother died when he was around 3 years old, but fortunately, his father remarried one of Eleanor’s close friends shortly afterwards. This wonderful woman (Lucille Marmon, from Texas) was the woman whom Carl’s children knew as their grandmother, and a wonderful mother to Eleanor and Con’s three children Carl, Michael, and sister Peggy. They would later be joined by John, Con and Lucille’s only child together, forming the Carrano “gang” of four.

Carl and his siblings grew up on Long Island (mostly Levittown)–a very different place from where he lived afterwards in California, Vermont and Texas. At that time, living in Long Island was to experience the golden age of American suburbia. Carl’s interest in chemistry started early. It was an age where every six-year-old child was given a chemistry set, but also when chemistry shows were common–and also quite dangerous. For these things, Carl and his brother were partners in crime. Carl and his brother Michael (Mike), one and a half years younger, would send their mother off to pharmacies with a shopping list of chemicals. Mike, Carl’s sort of second in command, tagged along gladly, doing his brother’s bidding–even when it turned out to be a hazardous occupation! Carl and Mike basically grew up blowing things up, putting mild explosives into drainpipes and generally being little terrors in the neighborhood. According to Mike, their parents were “torn between not wanting to suppress our self-education and not wanting us to burn the house down.” When Carl used an alcohol burner inside without permission and nearly burned the house down, their father finally cracked down on some of these activities, after which Carl was banished to an outdoor metal shed. While all this may sound extraordinary nowadays, these were normal 1950s boys’ activities. This was the golden age of “modern science”, with all its promise and innocence. There was a moon to be landed on in a few years, after all!

It was pretty clear from the beginning that Carl had a weak spot for chemistry, and that this is what he would pursue professionally. Carl graduated from Island Trees High School in Levittown on Long Island in 1968. From there, he went to the University of California, Santa Barbara (UCSB). This was a troubled time in the USA and on American university campuses. While at UCSB, Carl lived in Isla Vista, where he witnessed the Bank of America burn down after being set on fire by a student mob. Also, it was quite possible that he could have been called up to Vietnam, since there was still a draft. Carl told his family that there was a macabre sense of humor among the roommates when one of them was called up, recalling that they made him like a little cake in the shape of a tombstone. It is striking to think how Carl’s life could have taken a completely different turn if he had been called up for service in Vietnam.

Carl’s first research experience was with Bill Kaska, doing undergraduate research with him in his last year of study at UCSB (interestingly, the Carranos last saw the Kaskas during a *Queen Mary 2* crossing to Europe where they happened to be on the same boat!). At UCSB, Carl also knew Glyn Pritchard (+ July 8, 2020), who happened to become a friend of the author. Carl graduated with a B.S. (honors) in Chemistry from UCSB in 1972.

One of Carl’s lifelong friends, Dennis Mitchell, was a chemistry graduate student while Carl was an undergraduate at UCSB. Dennis’s own career took him around the world, including to Venezuela and France, but he and Carl always kept up contact, except for a four-year period while Dennis was in Venezuela. Fortuitously, they met again at Berkeley in the chemistry building elevator, both doing post-doctoral research there. Indeed, the author and Dennis have been the Carrano’s houseguests in nearly every place they have lived! Ellie says that it is Dennis and the author who have most faithfully followed them all over the world—that in fact they can’t get rid of us! The family has always speculated on what a meeting between Dennis and the author might be like. Food for thought.

After UCSB, Carl went to Texas A&M, where he earned his PhD in 1976 with Minoru Tsutsui, working on unusual metalloporphyrins as potential tumor-localizing agents (Tsutsui et al. 1975; Carrano and Tsutsui 1977a, b; Carrano et al. 1977, 1978; Austen et al. 1978; Mangani et al. 1983). Carl’s time in Texas was significant personally as well as professionally, as this is where Carl met Mary Watson (Mary was studying for her Masters in Chemistry at A&M). Mary, affectionately called “Toad”, would become his wife in 1976.

After doing his PhD at Texas A&M, Carl moved to UC Berkeley as a postdoc with Ken Raymond from 1977–79, which was a highly productive time. Here, he started working closely with Wesley Harris. After UC Berkeley, Carl moved to the University of Vermont, Burlington, as an assistant professor, where he got his tenure. Carl’s first daughter Jessica was born in Berkeley, while his second daughter Julia was born in Vermont, and his third daughter Eleanor (Ellie) was born in Texas, like his wife Mary (who grew up in Louisiana and Texas).

The Carranos stayed in Vermont for eight years, 1979–87. Those were very hard years for Carl and Mary in general, but especially for Mary, who was very lonely for a lot of the time in a tiny house in the middle of nowhere in Vermont. Carl, attempting to get tenure, was under a huge amount of stress and basically worked seven days a week. In hindsight, it seems like a real bifurcation in the road, another point at which the Carrano family’s life could have gone off in a different direction, a point at which Carl could have done something very different and left academia. Yet Carl overcame these challenges (including the setback of initially being unfairly denied tenure due to internal university politics, a decision which was later reversed), all with the extraordinary perseverance that characterized his life and academic career. His family remembers an incident which illustrates the kind of person Carl was. The day Carl arrived home with the heavy news that he had not gotten tenure, Mary reminded him of a carpooling arrangement they had made with an elderly woman of their acquaintance. In his distraction, Carl had totally forgotten to pick her up. Instead of capitulating to self-pity and simply neglecting his duty, Carl got back in the car and drove an hour or so back in the dark and snow to pick her up.

And that is the person that Carl was. He made a habit of doing the right thing, even when it was inconvenient. At the point that his character was tested, he got back in the car for that elderly woman, although it was the last thing on his mind. Carl was also totally committed to friendships and collaborations, and he would not let people down.

After 8 years in Vermont, the Carranos moved to Texas in 1988. After several job interviews in Texas (including one that frustratingly fell through at the last moment), Carl eventually took a position at Southwest Texas State University at San Marcos (which is now Texas State University, San Marcos), where he served as chair from 1988 to 1999. Eleanor was born in Texas in 1993. These were formative years for the Carrano family, as Carl built his career and the family enjoyed proximity to Mary’s family in Texas.

The family’s last move was to California, after Carl’s appointment to the position of Chair of the Department of Chemistry at SDSU in 2003. His move back to California was in part motivated by Carl’s love for the sea—he wanted to be closer to the ocean, and he had such good memories of going to Santa Barbara. After accepting the position in San Diego, however, Carl quickly realized (like everyone else in the early 2000s) that you have to keep moving further east until you can afford a house, and that that house is not going to be beachfront property in La Jolla. For the Carranos, it ended up being in the inland town of Ramona. The weather changes quite quickly as you go east from San Diego—it gets brutally hot, with a greatly increased risk of wildfires. The year after the Carranos arrived there, in late 2003, their second house almost burned down within a week after moving in, and again in 2007. The author visited just the weeks after this traumatic fire in early 2004—the fire stopped just feet from the wall of the house, visible by the burnt vegetation on the ground. It was shocking to see.

Living in Ramona was a love-hate relationship. Carl always said California was too screwed up, and that at some point he would take his money and move away. But everything's greener on the other side, and Carl had a well-known tendency to come to hate every place he lived in and to want to move on to the next. With hindsight, Ellie thinks that they had some very good moments in California, which included travel to Europe – including two family trips to Scotland in 2005 and 2007. She says Ramona was a wonderful place to grow up in and cherishes the years her family had there together.

It is remarkable that despite being Chair of Department, Carl remained research active and managed to go to the lab every day, even though on the management side of his job, he brought the department out of a bad financial situation at the time of the California budget crisis when he came in late 2003. This reflects his sort of brilliance—he combined administrative skill with being a world class scientist. This was at the core of the relationship and collaboration with the author. Carl’s professional modesty was on display again when he lost the position of chair, a move which was widely seen as totally inappropriate and unfair. Instead of complaining and holding grudges, Carl simply accepted the new situation and refocused on the opportunities this change presented him for renewed involvement in research activities.

Carl’s work ethic was extraordinary. Carl would take Christmas and Easter day off, but he used to work at home during other holidays or over longer breaks. Even later in his career, he would go in to work on Saturdays or come home late on weekdays. And yet none of this ever seemed to stem from an inordinate desire to “advance his career”, make more money, or avoid domestic life. Rather, it stemmed from a genuine sense of responsibility and a passion for his work. Carl also remained a steadfast practicing Catholic throughout his life, being active as a member of the Knights of Columbus at the Carranos' parish in Ramona. Being a professor never meant he was too good to put on an apron and wash dishes after a parish dinner or serve in countless other humble ways. Carl believed deeply in the complementarity of faith and reason, and combined a powerful intellect with a childlike trust in God.

In contrast to many academics for whom careers are about self-aggrandizement, Carl was very humble and modest. He cared more about the actual science than racking up meetings and conferences. Always thrifty and self-reliant, Carl was never too good for things. He would fix things in his own lab with a soldering iron, an art practically unknown to newer generation of scientists. He would talk about the fact that at least when he came to SDSU in the early 2000s, there was a dedicated facilities equipment person whom you could call in to fix instruments. Later on, they got rid of those positions, and if something broke, you just got rid of this $500,000 thing and you saved money on a salary that might have been $30,000. This was all part of the same trend of throwaway culture which Carl disliked. Similarly, and not surprisingly, Carl was very good at fixing things around the house. Together with his wife Mary’s complementary engineering mindset, the Carranos did nearly all the practical work (including a great deal of hard physical labor) around their California home. With wildfires a constant threat (nearly burning their home down twice), vegetation clearing was a constant battle that they undertook with minimal outside assistance.

In the lab, Carl was in his element completely. At some point Ellie asked him to do a distillation because she was in a phase of being obsessed with medicinal plants, which she is still very interested in. She wanted to do a distillation of spearmint leaves and get the essential oils. There was quite a bit of it all over the walls when she was done with it because of a small explosion of the distillation equipment. Ellie remembers that Carl did not hold back his opinion of this green disaster. Together with her mother, Ellie really lived much of her life in that lab, which became an important part of her childhood. Ellie feels that it was an incredible privilege to spend prolonged periods in the lab with her parents. The lab was in many ways the sort of Ellie’s and her parents’ second home. As Ellie recalls, if you needed a container or whatever at home, you would go in and look for it in the lab. Around half of the food containers at home somehow came from the lab—shampoo was put in Falcon tubes. Mary, for her part, worked faithfully for Carl in the lab (most of the time simply as a wife and volunteer), using her own educational background (Masters in Chemistry) and great knack for lab work. Eleanor recalls with great fondness the years during which the whole family was together at SDSU (Eleanor studying for her graduate degree in geology there): she would be doing homework in the little lab office, Mary would be in the lab, and Carl would be in his office just a minute away.

Things became blurred with the COVID-19 pandemic (which was particularly hard for Mary, since it meant the end of the whole lab experience for her) about 2 years before Carl passed away. Emotionally, the lab was so much part of life. And suddenly when Ellie’s father and Mary’s husband was gone, the lab was gone too.

Since summer 2021, Carl suffered from back pain, which came out of nowhere. Despite increasingly restraining his activities, and despite the absence of an obvious cause like an injury, things became worse, leaving him mostly bedridden by the end of the year. Around mid-January 2022, doctors understood that he was suffering from advanced multiple myeloma, from complications of which he suddenly passed away in the early hours of January 26.

Among the best relationships that Carl’s legacy has left the author with is his friendship and collaboration with several of the authors of this paper, including Eric Miller and more recently Andy Cooksy, with whom the author started interacting very nicely. He actually gave the author things he salvaged from Carl’s papers – including a painting that Ellie did probably when she was about 6 or 8 years old. The author subsequently visited Andy when he was on sabbatical at MIT in Cambridge, Massachusetts in January 2024, where they had a very nice day and a half together and salvaged an article on aerobactin which was initiated by Carl (included in this issue; Gardner et al. 2024).

For me, Carl was one of my closest friends and an excellent collaborator. I first met him when returning to Alison Butler’s lab (where I was an NIH-funded postdoc at the time) at the University of California, Santa Barbara, in mid-June 2002, coming back from a trip to the inaugural Gordon Research Conference Environmental Bioinorganic Chemistry in Andover NH and then a road trip to Cape Cod, New Hampshire, Maine and Massachusetts. At the time, Carl was again on a summer sabbatical in Alison’s lab, accompanied by Mary and their youngest daughter Eleanor (Ellie). Carl, Mary and I were working very long hours growing marine bacteria and isolating siderophores by HPLC, in particular aerobactin and petrobactin, which resulted in our papers on aerobactin photochemistry (Küpper et al. 2006) and petrobactin sulfonate (Hickford et al. 2004). The long working hours and the Carranos’ passion for Europe laid the groundwork for our friendship which lasts to this day. Besides successful science, highlights of that summer 2002 included our whale watching trip to the Santa Barbara Channel Islands (with sightings of 4 blue whales, several humpbacks, and hundreds of dolphins) and my rescue of cat Dorcas in front of their summer home rented from absent UCSB faculty in Isla Vista. I visited them in their new workplace in San Diego and home in Ramona in April and October 2003 – the latter was just days before moving to Scotland on 1 November, where I had been offered a lectureship and the post of Head of the Culture Collection of Algae and Protozoa at the Scottish Association for Marine Science (SAMS). Frequent, almost annual, mutual trans-Atlantic visits followed. Thankfully I had a great supporter for these activities in the late SAMS Director, Graham B. Shimmield, who had brought me from California to Scotland in 2003, who admired Carl and liked our research (as a marine geochemist), allowing me repeated sojourns in San Diego. I managed to visit San Diego in early 2004, 2005, 2006, 2008, 2009, 2010, 2014, 2015, late 2016, and a long spring break in 2018 which sadly was the last of my visits to Carl’s lab. I had already booked the tickets for a return in spring 2020, but that was cancelled due to the COVID19 outbreak. As I was looking into a return in early 2022, Carl’s health deteriorated rapidly and then shockingly passed away rapidly.

Reciprocally, the Carranos visited me in Oban, Scotland, in the summers of 2005 and 2007, and in Aberdeen (where he gave an invited lecture for the Royal Society of Chemistry) in late summer 2019. From there, we went to Oban for the Laminaria / iodine speciation diving work (Carrano et al. 2021). The Carranos fondly loved Oban and the West Highlands of Scotland. During the 2007 trip, Carl and went snorkeling with a pack of basking sharks off the Trotternish Peninsula, Isle of Skye. In 2015, I visited Carl and Mary during their sabbatical at the University of Kaiserslautern, Germany. These mutual trans-Atlantic visits greatly bolstered our research collaboration and clearly became a part of life for both of us and our loved ones. This included my late mother, who visited San Diego and Ramona three times and who befriended Carl and Mary, after having developed a fondness for California during my years as a postdoc in Santa Barbara. It also built upon Carl’s existing, strong ties to the UK scientific community (in recognition of which he was elected as a Fellow of the Royal Society of Chemistry in 2016, after having been elected as a Fellow in Chemistry by the AAAS in the USA in 2011).

I fondly remember not only Carl’s collaboration and scientific guidance for anything (bio)inorganic, but also the good times in their home in Ramona where I usually stayed with the Carrano family. I associate these times with Mary’s wonderful home-cooked dinners, usually preceded by a beer or an ouzo (which I had brought from Greece), swimming in the pool, the wilderness of the surrounding chapparal (Carl wanted “no neighbors” and had therefore chosen a house “in the bush”) with lots of cottontail rabbits and howling coyotes at night. During the weekends, I would hang out with Carl’s students in Pacific Beach, San Diego – in particular, with Eric Miller and Teresa Tymon –, where our activities included scuba diving, snorkeling with sea lions in La Jolla Cove, self-cooked dinners and parties.

My friendship with the Carrano family lives on and is now going into the next generation. In summer 2023, I visited Mary and Ellie in Ramona, which was to be my last visit there since Mary sold the house in late 2024 for moving closer to her daughter Jessica and her family in Virginia. In early August 2024, Mary and Ellie Carrano, Eric Miller and family and I had a memorial reunion for Carl in Eric’s home in Tysons Corner, Virginia. In recent years, I have developed a friendship with Carl’s youngest daughter Ellie who assisted teaching my international field course (run jointly with Dr. Vasilis Louca at the University of Aberdeen) “Mediterranean Marine Biology” in Nea Peramos, Kavala, Greece, in late May 2022. After Ellie moved to Kuwait to become a science teacher at the American Baccalaureate School, I visited her and my former PhD students Amal Hajiya Hasan and Hanan Al-Adilah during a very memorable 10 days in Kuwait in April 2024 which included scuba diving at Qaruh Island in the Gulf (an important study site of Amal’s PhD in 2019; Hasan et al. 2023), road trips of the Kuwaiti desert and, which, significantly, in Amal’s holiday home in Khiran close to the Saudi border, provided the setting for my interviews with Ellie upon which this article is founded. In summer 2024 and motivated by Ellie’s passion for the Middle East and the Palestine cause, I introduced her to my long-time friend Ambassador Mark G. Hambley, veteran of the State Department and former U.S. Ambassador to Qatar and Lebanon, and his wife Patricia, now retired in Florida, with whom we spent several days during a road trip to the Deep South of the USA. On the same occasion, I befriended Carl’s brother John and his wife Nancy during 2 stopovers in their home in South Carolina.

**Fig. 1:**
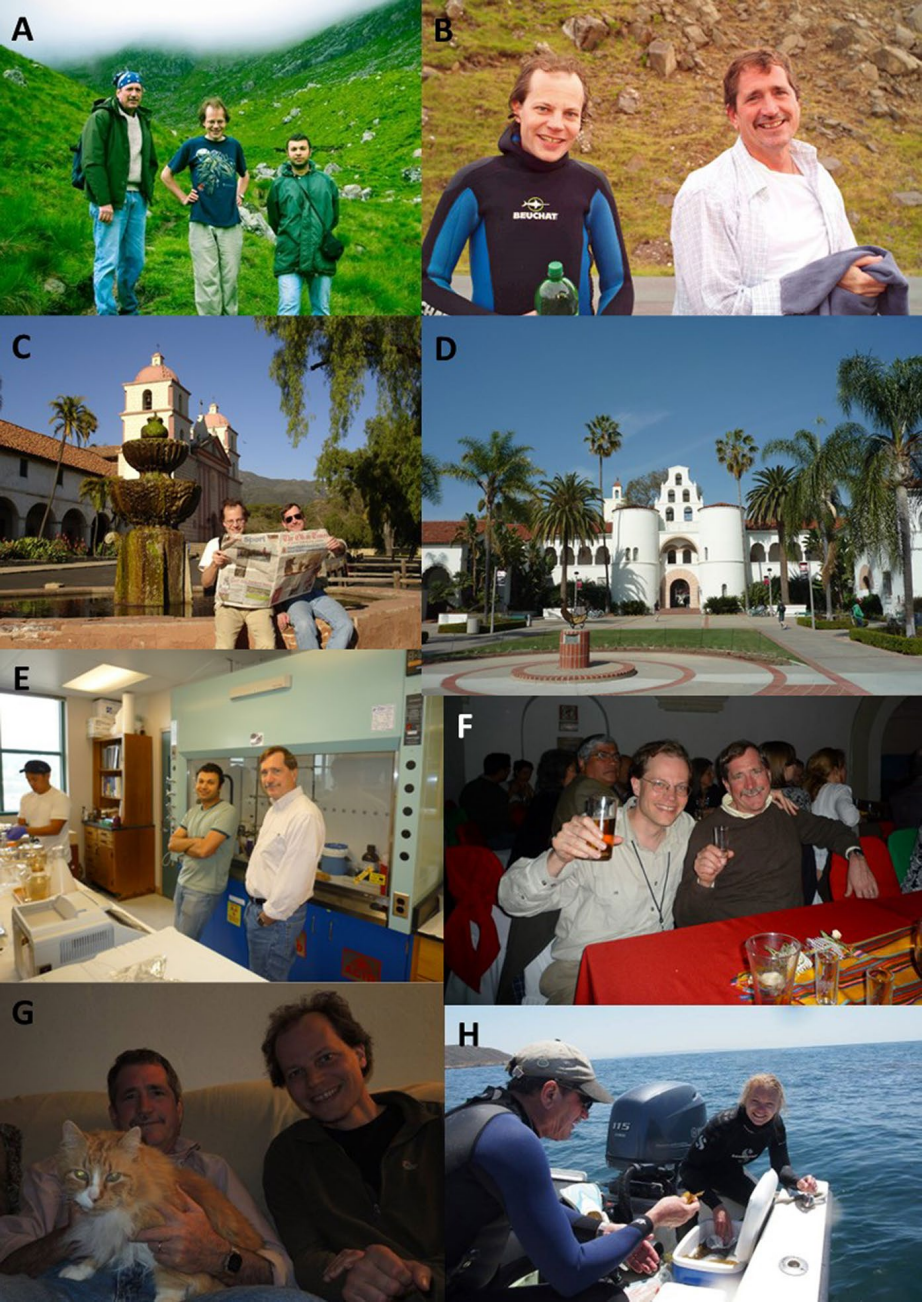
**A.** Carl, Frithjof, and Shady Amin hiking near Oban, Scotland, Summer 2008. **B.** Carl and Frithjof after their basking shark adventure off the Isle of Scotland, Scotland, 22 July 2007. **C.** At the Gordon Research Conference Metals in Biology, Jan. 2009. **D.** San Diego State University, Feb. 2010. **E.** With Shady Amin in the lab, Feb. 2010. **F.** With Frithjof at the International Seaweed Symposium in Ensenada, Feb. 2010. **G.** Carl, Dorcas the cat and Frithjof in Ramona, April 2015. **H.** Carl and Teresa Tymon sampling seawater near Point Loma for iodine speciation analysis, April 2015

**Fig. 2:**
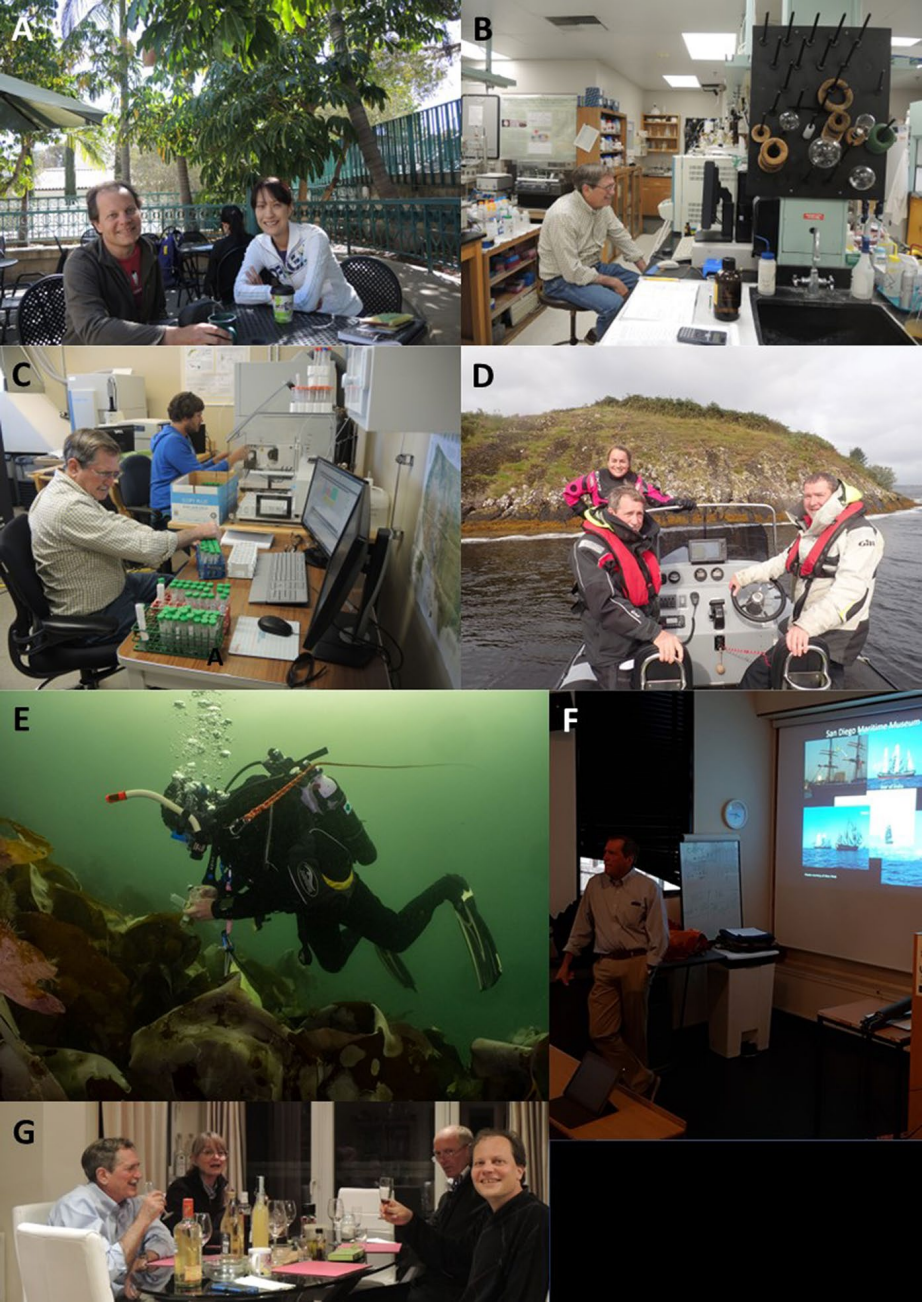
**A.** Frithjof with Carl’s former PhD student Kyoko Yarimizu at SDSU, April 2018. **B.** Carl in his lab at SDSU, March 2018. **C.** Carl doing ICP-MS at Scripps Institution of Oceanography, April 2018. **D.** Carl (front left) on a dive boat of Tritonia Scientific with Joanna Smart (back) and Hugh Brown (right) and **E.** Frithjof collecting seawater samples by syringe following a technique developed by him and Carl near Oban, Scotland in September 2019. **F.** Carl during his Royal Society of Chemistry lecture at the University of Aberdeen, September 2019. **G.** Dinner in Frithjof’s home in Aberdeenshire, Sept. 2019: Carl, Mary, visiting phycologist Akira F. Peters and Frithjof

**Fig. 3:**
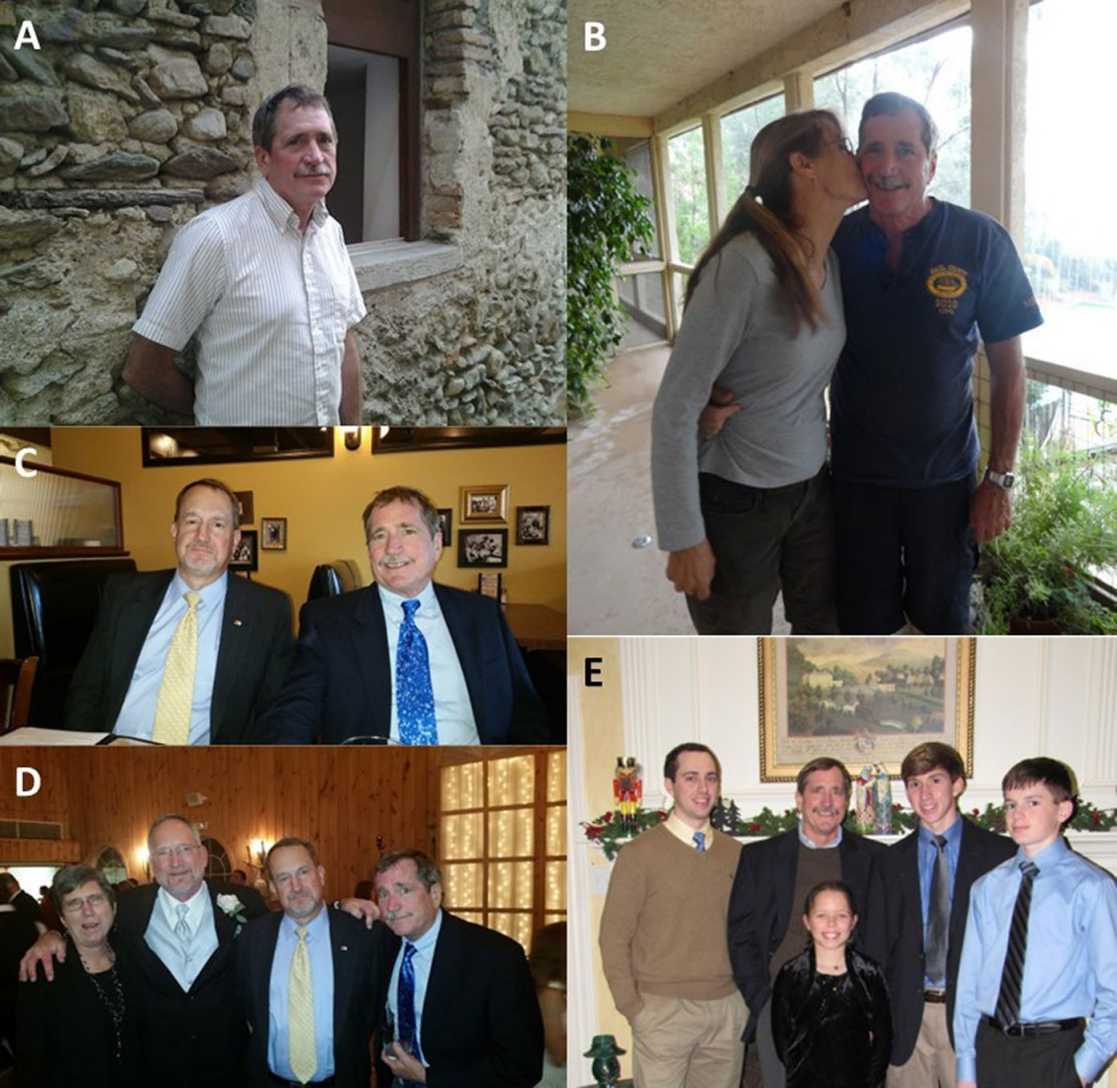
**A.** Carl in Lugano, Switzerland on 9 July 2015 (photo by Mary Carrano). **B.** Mary and Carl on the veranda of their house in Ramona, 25 July 2014. **C.** John and Carl in Connecticut (for their niece’s wedding), 14 November 2015. **D.** Peggy, Michael, John and Carl (the 4 siblings) Connecticut (at their niece’s wedding), 14 November 2015. November 2015. **E.** Kids with Uncle Carl in Austin, Texas, 24 December 2011. (B-E from John Carrano)

Scientifically, Carl was certainly a key collaborator of my entire scientific career for which I will remain eternally grateful. Initially, he has played a central role in my research on siderophores of marine bacteria, which started during my time as Alison Butler’s postdoc at UCSB in 2001–2003 where he was a great mentor in a chemistry lab (Hickford et al. 2004; Küpper et al. 2006; Zhang et al. 2009) after graduating with degrees in biology. This continued and intensified after my move to SAMS in Scotland when starting to collaborate with David H. Green who had isolated numerous bacterial strains which were symbionts of harmful algal bloom microalgae (chiefly dinoflagellates). This resulted in the discovery of boron binding to siderophores (Amin et al. 2007; Harris et al. 2007) and the role of bacterial siderophore photochemistry in facilitating algal iron acquisition (Amin et al. 2009a, 2009b), triggering our interest in boron as an unusual bioelement (Carrano et al. 2009). Since 2007, we increasingly moved the focus of our collaboration from bacterial to algal systems. Together, we participated in the annotation of the first seaweed genome to be sequenced, of the filamentous brown alga *Ectocarpus siliculosus*, covering bioinorganic (especially halogen and iron) metabolism (Cock et al. 2010, 2012). A key finding was the absence of ferritin in brown algae, subsequently confirmed by physical and biochemical approaches (Böttger et al. 2012; Hartnett et al. 2012a, b; Miller et al. 2014). An unforgettable trip south of the border took us to Ensenada, Baja California Norte, Mexico, for the International Seaweed Symposium in late February 2010. Since about 2013, Carl became involved in my long-standing research on algal halogen metabolism. In our collaboration, we focused on Pacific algae (chiefly giant kelp, *Macrocystis pyrifera*), establishing key features of its iodine metabolism (Tymon et al. 2017). Based upon an expedition to Chile in 2014, we investigated key features of the halogen and arsenic metabolism of 3 large southern hemisphere brown algae, *Durvillaea antarctica*, *Macrocystis pyrifera* and *Lessonia trabeculata* (Miller et al. 2025). Due to Carl’s passion for oceanography, we subsequently started investigating the role of seaweeds in the iodine speciation of coastal waters, which involved a lot of boat-based work for Carl and scuba diving for me and some of Carl’s students, respectively. This showed that the iodide / iodate ratio is strongly associated with the presence of giant kelp biomass (Gonzales et al. 2017; Carrano et al. 2020). We subsequently made similar findings with European *Laminaria* kelp species on the coast of Scotland and France (which turned out to be Carl's and Mary's last joined trip to Europe; Carrano et al. 2021), showing that they increase iodide levels in coastal waters when exposed to oxidative stress, corresponding to the antioxidant role of iodide as antioxidant in these organisms (Küpper et al. 2008, 2011, 2013). When investigating iodine metabolism in *E. siliculosus*, we found that also a non-haloperoxidase pathway is at work in the synthesis of iodomethane (Küpper et al. 2018; this paper was completed during downtime on an expedition to Rothera, Antarctica). In more recent years, we became interested in the cellular storage mechanisms and speciation of iodine, bromine and trace metals in brown algae, for which we conducted X-ray tomography at the European Synchrotron Radiation Facility (ESRF) in Grenoble in June 2018 and micro-XANES at the Advanced Photon Source in Argonne, Illinois, in October 2018, which involved collaboration with my friend, the algal cell biologist Christos Katsaros at the University of Athens, Greece, resulting in the discovery of a hitherto undescribed type of vesicles in brown algal cells with electron-dense content which may correspond to iodine (Katsaros et al. 2021). The combination of tomography and micro-XANES proved a powerful tool to combine chemical speciation with subcellular speciation (Mijovilovich et al. 2023). Our work on algal halogen biochemistry also contributed 2 review papers (Küpper and Carrano 2019; Al-Adilah et al. 2022) (Figs. 1, 2, 3).

## Ken Raymond

*Farewell, dear Carl*

*Carrano Comments*

**Fig. 4:**
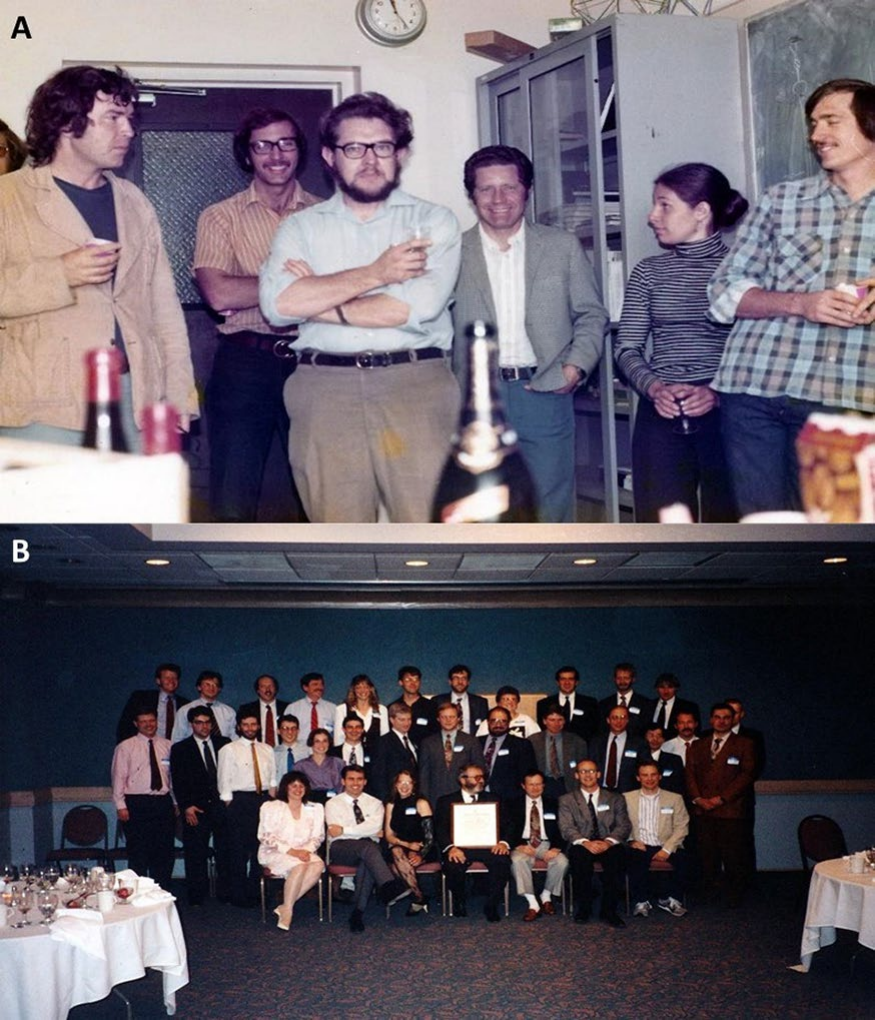
**A.** Carl (right) with members of Ken Raymond’s lab at Lawrence Berkeley National Laboratory (LBNL) in the 1970s. **B.** Carl (fourth from left at top) at Ken Raymond’s American Chemical Society Alfred Bader Award in Bioinorganic or Bioorganic Chemistry (New Orleans, 1994). (Images from Ken Raymond)

Carl joined my lab in 1977 following his Ph. D. research and brief postdoc on metalloporphyrins with Minoru Tsutsui at Texas A&M University. This was in the early days of my siderophore (microbial iron transport agents) research, which Carl played a very prominent role in beginning. Early on I realized that relative stability of these compounds and other labile biological iron complexes would play a key role in their function and so I learned how to perform the experiments needed to determine this, primarily from the works of Arthur Martell (whom I regard as my correspondence teacher on this subject). I do not recall if Martell (who was also at A& M) recommended Carl, but you will see that Carl participated in, and led, several of the first of these endeavors, which required developing the hardware and computer software for such experiments. Carl was one of my most brilliant and productive postdocs at perhaps the most productive time of my career. So many of the things he did with me came more from his brain than mine. The characterization of the di-hydroxamate siderophore Rhodotorulic Acid (Carrano and Raymond 1978a, c) were the first of his papers with me followed by this siderophore’s role in iron uptake in *Rhodotorula pilimanae* (Carrano and Raymond 1978b) and the solution equilibria and electrochemistry thermodynamics of ferric Rhodotorulate complexes (Carrano et al. 1979). He later played a similar role in the isolation, characterization, and thermodynamic properties of another di-hydroxamate siderophore, Ferric Aerobactin (Harris et al. 1979b). Carl also played a key role in determining the stability constants and electrochemical behavior of Ferric Enterobactin, the tricatcholate siderophore with the highest known affinity for ferric ion (Harris et al. 1979a, 1979c), and the kinetics and mechanism of this siderophore’s iron removal from the human iron transport protein Transferrin (Carrano and Raymond 1979). Carl also co-authored reviews and several other papers on other topics in my lab at the time. When Carl left my group, I would have supported any application he made to the top universities in the country, but he was modest in his plans and expectations. He was a gentleman and gracious as well as a demanding scientist with high standards (Fig. 4).

## Eric Miller

*In Memoriam of Carl Carrano.*

*July 7, 2024.*

Carl Carrano was my PhD advisor from 2010—2015. It is often said that choosing your advisor is one of the most important decisions one can make in their college career. During my rounds as a first-year Master’s student, I found this decision to be obvious: Carl was well-funded, a prolific author, and conducted multidisciplinary research which complimented my educational background. I recall, at the time, being surprised that no other prospective grad students were considering joining Carl’s lab. Later, I realized that Carl was simply not the type to pander to an audience. He was direct and no-nonsense, but also easy-going and friendly. His style was always appropriate to the context of the conversation at hand. As Chair of the Chemistry Department, he had a big responsibility and all the stress that came along with it. With the department facing major challenges at the time, he had to communicate tough topics and make tough decisions. For example, at our incoming grad student orientation meeting, Carl made an impromptu entrance holding a pair of safety glasses. He held them up so that everyone could see that the lens had projectile material embedded in it. He made it crystal clear that if he observed anyone not wearing personal protective equipment in the lab, our graduate careers would be over. I remember the other students recoiling in fear after Carl exited. I was then reminded of the generational gap separating myself (GenX) with the others (Millenials). Not only did I receive the message loud and clear, but I also really liked the style in which he did it. Perhaps it was eight stark years of real-world experience that had thickened my skin. The following five years as Carl’s mentee were extremely pleasant and productive; halfway through my Master’s it was clear to us that the PhD program was a promising path. He encouraged me to take the lead on authoring manuscripts just two years into the program, paving the way for a timely dissertation. Carl loved the opportunity to escape administrative headaches and get back to the bench; he was often in the lab troubleshooting ornery instruments, exploring new analytical techniques, and overseeing a very clean & efficient lab. When Mary and Ellie started volunteering in the lab, it provided a nice opportunity to witness the Carrano family’s love and harmony, albeit cloaked in terse professionalism. Still, after introducing the lab group to his family, most of us referred to him as “Dr. Carrano”. The topic came up at least once and he explained, “I’ve offered many times for them to call me Carl, but they keep calling me Dr. Carrano. So, I got tired of repeating myself and just let them call me Dr. Carrano.” For those who know Carl well enough not to fear him, hold a deep respect and admiration for him.

Carl’s diverse collaborations were instrumental to my success and development as a scientist. Many vibrant scientists visited Carl’s laboratory, inspiring me to broaden my intellectual horizons. These collaborations, combined with Carl’s grant awards, allowed me to travel twice to a synchrotron facility and thrice to Europe. While in Europe, I met our German colleagues Berthold Matzanke, Volker Schünemann, and Frithjof Küpper. Berthold and Volker were wonderful hosts while Frithjof continues to be a great friend and colleague. Frithjof and I have rendezvoused numerous times during our travels in the U.S. and Europe, always reminiscing of Carl and sharing fond memories of our friend who passed too soon. Carl, thank you for being such a genuinely kind, supportive, and impactful mentor. You are dearly missed.

## Ricardo Cruz-López

My first contact with Carl was when I read his paper on iron siderophores in PNAS, which intrigued me about this iron interaction between bacteria and phytoplankton, as I was working on a similar mechanism for my PhD at the time, but with vitamins. It was 2013 and the paper had been published for a while. After some thought, I sent him an email asking for a strain of bacteria that he had used in that paper, as I was working on a fluorescence in situ hybridization protocol for bacteria associated with dinoflagellates. I was surprised when he emailed me back a few days later, inviting me to come to his lab and pick up, which I accepted as I was not even 100 miles away, but in a Mexico. I took the bus from Ensenada to Tijuana, crossed the border, took the trolley from Chula Vista to the SDSU station, and finally got to his lab. We talked for an hour, while he showed me his lab, and I think I only met Eric Miller, one of his graduate students, I don´t remember that well. I went back to Ensenada the same way, trying to avoid the all the traffic. Back in Ensenada, I started working with the strain as a positive control for my FISH-side project, and in a couple of months I had enough results and emailed him about my progress and ended up with similar results to one of his master´s students, but a different approach. Since I was trying to finish my thesis, I had to focus more on it and for a few months I had no communication. The graduation came, I started to email him again, looking for some way to continue with the collaboration, and it was during these days that I met Kyoko, one of his PhD students. Since Carl was doing some visiting or sabbatical in Germany, he introduced me to Kyoko through email, and in some funny way Kyoko started coming to Ensenada to learn some culture techniques about phytoplankton. From time to time, Kyoko crossed the border to come to the lab, not only to continue learning these techniques, but also to enjoy the seafood of the region, which I also enjoyed. Much has happened in that time, and Kyoko and I continue to work together and be friends. I remember the cruise we went on to track harmful algal blooms together with colleagues from Ensenada, this cruise lasted about three days, Kyoko got really sick due to the weather, and this is something Carl took as a nice adventure for Kyoko… coming to Mexico with only a few of days to prepare the material, crossing the border, in a last call, almost not making it given the Mexican Sea regulations, but in the end we made it! Another time passed, and through a Mexican postdoctoral fellowship I was able to go to SDSU and work directly in Carl´s Lab.

## Ana Mijovilovich

*A spectroscopist’s delight: account of a dream match of Chemistry and Spectroscopy.*

For more than two years during the pandemic we had a number of online meetings to work out the last algae data taken at two synchrotrons. Those were very dense and lovely chemistry discussions, among all well-seasoned and high-ranking Chemistry and Biology professors, and one woman: me.

In the science Carl was not quick in jumping to conclusions. He would lean back in his chair and come with a number a question, after seeing good looking data fits. Now science is collaborative and interdisciplinary, and the ability to talk to others with different background is essential. Carl excelled as a listener. And he was a “humble” listener, in spite of his gigantic h-index (h = 51 by June 2024 at WOS). This contributed to a very collegial environment during scientific discussions. The zoom meetings were pure fun, just a bunch of people who love chemistry and algae.

Carl was a facilitator sharing all kinds of old data and former collaborations together. He communicated what he knew but also what he did not, making the questions that led to the next synchrotron measurements. Being close to retirement, and in a multi-authored paper with many high-profile professors, instead of the “last glory trumpets” for himself regarding his position in the authors list, he recognized my contribution with one of the main author positions at the stage of drafting the paper.

Carl worked as early as 1978 on Fe uptake by siderophores in microbes (Carrano and Raymond 1978a), in 1984 he used Mössbauer spectroscopy to characterize Fe in model compounds (Carrano and Spartalian 1984), and he started studying fungal ferritin in 1996 (Carrano et al. 1996). Ferritin was first characterized with Mössbauer spectroscopy by Boas and Window in 1966 (Boas and Window 1966) and later studied in many organisms by Erika Bauminger (38 references in WOS). In algal studies, a WOS search “ferritin AND Mössbauer AND algae” gives only two papers, both from Carrano in collaboration with spectroscopists and biologists (Böttger et al. 2012; Hartnett et al. 2012b). By combining both Mössbauer and synchrotron XAS, it was possible to determine a ferritin-like Fe mineral storage in *Ectocarpus siliculosus* and *Emiliania huxleyi,* both lacking ferritin encoding genes. Our “bio-chem-spectroscopy” collaboration took a step forward in his last work, by using microprobe and nanoprobe beamlines to determine the metal localization and binding at different tissues, this time in “intact frozen samples” (Mijovilovich et al. 2023).

Carl left at the dawn of even more powerful tools to analyze the algae with low emittance synchrotrons, free electron lasers and Machine Learning. These new analytical and theoretical tools bring amazing possibilities by reaching lower levels of detection and decoding more complex compositions. Carl’s contribution is not history but a living organism, and we will be always inspired by his pioneer work.

## Kyoko Yarimizu

*Associate Professor, Microbial Genomics and Ecology, The IDEC Institute, Hiroshima University.*

Inspired by Dr. Carrano’s innovative research, which sought to elucidate the algal–bacterial role in harmful algal blooms, I endeavored to become his student. From 2012 to 2018, I enrolled in the Ph.D. program under his mentorship. As Dr. Carrano’s final doctoral student, I had the privilege of receiving invaluable one-on-one guidance from him. Under his supervision, I completed my research project entitled “The Role of Iron in Potential Algal–Bacterial Mutualism as Related to Harmful Algal Blooms” in 2018.

Dr. Carrano’s expertise extended well beyond his original focus of study in Inorganic Chemistry, expanding into the intricacies of Biochemistry. His exceptional teaching, guidance, and writing skills made him an outstanding mentor. While I learned many things from him about research, he imparted to me, most importantly, confidence in myself amidst the setbacks of a long Ph.D. program. I vividly recall my second-year oral examination, a rigorous test defending my science against relentless questions from the thesis committee, which extended far beyond its intended duration. I even received a parking ticket for the extra hours spent on my defense. Upon returning to the laboratory, exhausted and in tears, Dr. Carrano congratulated me for passing the exam and emphasized the importance of moving on to the next phase. His belief in my abilities often surpassed my own. Dr. Carrano encouraged me to cultivate self-respect and assurance. Even now, in moments of doubt, his unwavering faith in my potential illuminates my path forward, providing reassurance and instilling confidence to navigate the complexities of my career.

While the loss of Dr. Carrano leaves a profound void within the academic community, his teachings and mentorship serve as a timeless legacy, inspiring future generations to emulate his unwavering commitment to the pursuit of knowledge. As I embark on my own scholarly endeavors, I carry with me the enduring lessons imparted by Dr. Carrano, forever grateful for the privilege of having walked alongside a true visionary in the realm of chemistry.

## Andrew Cooksy

*Professor, Department of Chemistry and Biochemistry, San Diego State University*

Carl’s first faculty appointment was at the University of Vermont, where he served for 8 years before being invited to become Department Chair at Southwest Texas State University. (SWT) from 1988 to 2000. After stepping down from that position, he learned of a nationwide search for a chair to lead the Department of Chemistry and Biochemistry at San Diego State University (SDSU), and he applied for the position. Tom Scott, Dean of Sciences at SDSU, found that Carl was “widely respected at SWT for building the research base at SWT while maintaining a strong undergraduate program. Faculty and administrators at SWT were unanimous in their praise for his scientific achievements and his managerial skills.” Carl joined the SDSU faculty as Chair in 2003. He came to SDSU with a recently renewed NSF grant, which he used partly to install a new powder x-ray diffractometer, significantly expanding the scope of the department’s analytical capabilities.

Carl captained SDSU Chemistry through its most difficult period in memory, taking the helm during California's long recovery from the dot-com bust of 2001 and holding fast through the Great Recession of 2008. In this difficult environment, he strove successfully to build relationships with alumni and other potential donors, establishing a foundation for future gifts to sustain the research and teaching programs.

Carl's leadership of the Department put a premium on insulating faculty from persistent administrative demands, which meant that he handled much more than his share of the day-to-day office workload. The faculty valued his forthrightness as well as his work ethic. In particular, he was appreciated for holding the upper administration to the same high standard he applied to himself and his colleagues, and he was unsparing in his criticism of administrative missteps. One representative email from Carl read, “In the stupidest thing since Nero fiddling while Rome burned, we have been given $175 K for new gizmos while we are laying off staff, have no operating or maintenance funds etc. Sheer lunacy.” Another email on the topic of faculty activity forms reads in part, “Understand that virtually everything outside of actual classroom assignments on these forms are in fact made up/fabricated to appease some bean counter somewhere. It has no meaning in reality! … I hope this will clear up the evident misunderstandings and allow us to get through a useless but time-consuming university requirement.”

Accompanying his clear-sighted leadership of the department was Carl's brilliant scholarship. In 2012, his research accomplishments led to Carl's election as a Fellow of the American Association for the Advancement of Science, only the sixth in the history of SDSU. His prior service as an NSF program officer made him a valuable resource to the department. Several younger faculty recall that meeting Carl was perhaps the most valuable experience of their hiring interview; he was so generous with his experience and advice. The Department staff also remember him with great affection, valuing the frankness that was his hallmark.

Carl left the chair’s position in 2013 and continued to carry out research and teach inorganic chemistry. He served the department several years in partial retirement, and was beginning his final semester at SDSU when he became ill.

## Wes Harris

Carl and I overlapped as graduate students at Texas A&M University, but I barely knew him at the time. I graduated a semester after Carl, and by a remarkable stroke of good fortune, I ended up occupying a desk next to him as a postdoc in Ken Raymond’s lab. We were both working on the siderophore project. Carl had already finished studies on rhodotorulic acid, so he and I (and several others) were focused on aerobactin and enterobactin, as well as synthetic analogs of enterobactin that were being synthesized in the Raymond lab. Working in the Raymond lab was an amazing experience. Ken was a great postdoctoral advisor/mentor, the projects were going well, and Carl was at the center of it. He was a brilliant colleague, hard-working and creative, generous with his time and ideas, and with a relaxing demeanor that made it a real pleasure to work with him. In addition to the work on siderophores, Carl and I started some work on metal binding affinities of the iron transport protein transferrin. We studied thorium binding while we were at Berkely (*J. Am. Chem. Soc. 1981, 103, 2231–2237*), and collaborated on a study of vanadyl and vanadate binding after we had left to take independent positions (*J. Inorg. Biochem. 1984, 22, 201–218*). Our last formal collaboration was in 2006, when I had the good fortune to join a study on the binding of borate to marine siderophores with Carl and Professors Shady Amin and Frithjof Küpper (*J. Am. Chem. Soc 2007, 129, 478–479* and *J. Am. Chem. Soc 2007, 129, 12,263–12,271*). Carl was a good friend, and one of the finest colleagues I had in my 40 + year career.

## Shady A. Amin

**Fig. 5:**
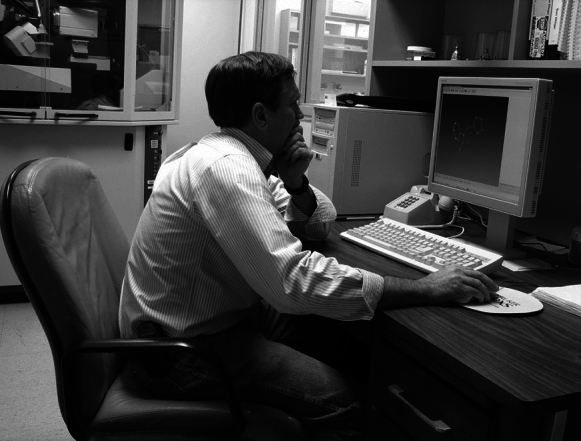
Carl at the X-ray crystallography instrument at SDSU (photo by Shady Amin, ca. 2009)

I had first met Carl in 2003 when I first joined the Chemistry Master’s program at San Diego State University (SDSU). Carl had just recently moved to San Diego from Texas at this point and I was one of the first graduate students to join his lab. I was inspired by his research on siderophores and particularly a new project examining the role of siderophores produced by some marine bacteria in providing bioavailable iron to phytoplankton in iron-limited environments. I continued working in Carl’s lab until I decided to apply to the joint PhD program in Chemistry between SDSU and UC San Diego under Carl’s supervision until 2010. During this time, I was fortunate enough to have learned, interacted, and engaged in many scholarly and personal conversations with Carl twice a weekday for a total period of seven years. I also built his lab’s website and in the process took a number of photos of Carl that as far as I know are the most recent photos of him in an academic setting. It is during this time that my experience as a chemist, and as a future oceanographer, matured. Under his supervision, I coauthored a total of 11 peer-reviewed publications, including in JACS and PNAS. I was also fortunate to have accompanied Carl to the Scottish Association of Marine Science for 3 summers during my graduate work to visit our collaborators Drs. Frithjof Küpper and David Green and conduct critical microbiology experiments related to my thesis. After leaving Carl’s lab in the summer of 2010, we kept in touch and continued to have occasional conversations either in person during my visits to San Diego, or online (Fig. 5).

## Alison Butler

I met Carl when I was a graduate student at UCSD in 1981 at a NATO Advanced Study Institute on "Coordination Chemistry Environments in Iron-Containing Proteins and Enzymes—Including Smaller Molecules and Model Systems" organized by Ken Raymond, Brian Dunford, David Dolphin and Larry Sieker. Carl was a postdoc in Ken’s lab at the time. This was a phenomenal conference, starting off at the University of Alberta and then moving to a lodge just outside of Jasper National Park in the Canadian Rockies. For the later part of the conference, as I remember, talks were in the morning and evening, with time in the afternoons to hike. One long and strenuous afternoon hike was most memorable, as I had forgotten my sack lunch, but Carl gave me his apple core for sustenance! Carl and I had interests in the chemistry of vanadium and iron. He suggested spending the summer of 1990 in my lab which turned out to be quite fortunate for my research group. He arrived in early July, just days after our house burned down in a large wildfire in Santa Barbara. My entire lab appreciated his interests in each person’s project. He returned again in the summers of 1992, 1997, and 2002, maybe as a respite from summers in Texas. During the last trip, Mary also worked in the lab, which was wonderful! Frithjof Kuepper was a postdoc in my lab then and Frithjof, Carl and Mary struck up an enormously successful working relationship and friendship which continues today. Carl’s and Mary’s youngest daughter, Eleanor, was between the ages of my daughters. The year after Carl’s and Mary’s latest visit we had the pleasure of welcoming Eleanor to stay with us so the kids could attend a UCSB Surf Camp. Carl and I had many similar scientific interests. He was a great collaborator. The students in my lab who interacted with him and I all benefitted enormously from his insights and comments.

## John C. Carrano

*Childhood and family memories.*

When my brother Carl was a young boy he received one of those “really cool” chemistry kits for Christmas. This was probably in the early 1960’s. I am quite sure Carl developed his love of chemistry with that fun science kit. By the time he was in high school, Carl was doing some rather sophisticated chemistry experiments including, unfortunately, making explosives. Since he was smart enough to know our father would kill him for making explosives, Carl cleverly hid the work in our attic crawl space – not really a good idea, but in the mind of an excited teenager, he thought it was the perfect solution; that is, until our father discovered it and nearly skinned him alive. Well, Carl and our Dad reached a compromise solution: Dad bought a shed for Carl and built it in the backyard where Carl could do his experiments. Carl actually made our 4th of July fireworks, which my Dad loved. Unfortunately, Carl did not stop there. He got the “great” idea of building a series of demolition charges that he daisy chained together. He had “carefully” tested these charges at an overflow reservoir near our home; so, he thought he had a pretty good idea of the power of the explosives he had made. Then came the fateful error: Carl decided it would be a great idea to place these charges under the manhole covers that went down the middle of our little suburban street. I think he placed about 4 charges under adjacent manhole covers. He then set them off with perfect timing; so, simultaneously four massive steel covers went flying in the air with huge flames shooting out of them. This was unfortunate since all the moms completely panicked thinking the neighborhood was about to go up in flames. The fire department came in and put out the flames and of course discovered this was the result of explosive charges. Carl was the prime suspect, but never charged! The good news is that Carl learned a very useful lesson about explosions in confined quarters. Hey, we all learn differently – for some of us it takes a near tragic outcome for the lesson to get pounded in our skulls.

A few years later, Carl was in his chemistry shed making soap. It was the weekend of the NFL Super Bowl and Carl loved football. So, he ran between the shed and our family room (where I was happily enjoying the game) so he could continue his soap experiment and catch the game. At one point, Carl went out to the shed to check on things. When he opened the door the boiling lye (or whatever it was) decided to explode in his face. I am guessing that by opening the door, the equilibrium conditions were sufficiently disturbed to cause the sudden explosion. I can remember quite clearly holding Carl down in the bathtub and my father desperately running water over his face and eyes. He suffered some burns, but his eyesight was just fine. Looking back on it all, I am not sure how my father survived having such a clever kid who was at the same time a complete knucklehead!!

After Carl received his doctorate in chemistry, and got his first position as a college professor, he returned to Long Island (our hometown) to visit his old high school chemistry teacher. I do not recall the teacher’s name, but I do vividly recall returning home and telling us how the man cried when he learned of Carl’s great success in chemistry. I am quite sure that was probably the defining moment for that nice high school teacher’s entire career. And Carl carried that attitude with him into his academic career. He loved teaching kids and was passionate about his profession. But Carl’s greatest joy came from doing research. He never worried much about his “status” or his “reputation;” rather he just wanted to do good work – which literally became his life’s work. And he did plenty of great work.

I also earned my doctorate and Carl attended my graduation. He was always a very good big brother to me – he was very proud that I had graduated from West Point, and that I served for a few decades in the Army. But, I think when I received my PhD that Carl was really, really proud of me – and that meant the world to me. I miss him greatly.

LTC (ret.) John C. Carrano, PhD.

Greenville, SC.

October 2nd, 2024.

## Data Availability

No datasets were generated or analysed during the current study.
